# Complete Genome Sequence of *Spiroplasma turonicum* Strain Tab4c^T^, a Parasite of a Horse Fly, *Haematopota* sp. (Diptera: Tabanidae)

**DOI:** 10.1128/genomeA.01367-15

**Published:** 2015-11-19

**Authors:** Robert E. Davis, Jonathan Shao, Yan Zhao, Gail E. Gasparich, Brady J. Gaynor, Nicole Donofrio

**Affiliations:** a Molecular Plant Pathology Laboratory, USDA, Agricultural Research Service, Beltsville, Maryland, USA; b Department of Biological Sciences, Towson University, Towson, Maryland, USA; c Department of Plant and Soil Sciences, University of Delaware, Newark, Delaware, USA

## Abstract

*Spiroplasma turonicum* was isolated from a *Haematopota* sp. fly in France. We report the nucleotide sequence of the circular chromosome of strain Tab4c^T^. The genome information will facilitate evolutionary studies of spiroplasmas, including symbionts of insects and ticks and pathogens of plants, insects, crustaceans, and humans.

## GENOME ANNOUNCEMENT

Although the helical, motile, and cell wall-less bacteria termed spiroplasmas were first discovered in plants, spiroplasmas have been isolated from diverse insects, ticks, and crustaceans (1–10). These discoveries and the identification of a human-infecting spiroplasma as a strain of the horse fly (*Haematopota* sp.) symbiont, *Spiroplasma turonicum* (11), support the prediction that spiroplasmas exhibit a constant link with arthropods (2).

Here, we report the complete nucleotide sequence of the circular chromosome of *S. turonicum* strain Tab4c^T^ (American Type Culture Collection, ATCC 700271), which was isolated in axenic culture from a *Haematopota* sp. fly in France (6). Genomic DNA was extracted from a culture of *S. turonicum* Tab4c^T^ grown in liquid M1D broth medium, as previously described (12). Nucleotide sequencing was carried out using the next-generation sequencing (NGS) platform of the Pacific Biosciences (PacBio) single-molecule real-time sequencing (SMRT) system, in which 86,887 reads were obtained, totaling 1,193,390,409 nucleotides. The *N*_50_ read length was 17,755 nucleotides, the mean read length was 13,734 nucleotides, and the average reference consensus concordance was 100.00%. The assembled circular chromosome of 1,261,374 bp has an overall base composition of 24.20 mol% G+C; the average coverage per base position was 616.05×. The assembled chromosome was put through GeneMark.hmm (13) annotation and was curated by manual inspection using Artemis (14) as an annotation platform. The programs tRNAscan-SE 1.21 and RNAmmer (15) were used to predict regions containing tRNAs and rRNAs, respectively. The chromosome has 1,085 protein coding regions (CDSs), 1 set of rRNA genes, 29 tRNA genes, and a 2,940-bp clustered regularly interspaced short palindromic repeat (CRISPR) locus.

Phylogenetically, *S. turonicum* clusters with other wall-less bacteria in the class *Mollicutes* and is a member of the Apis clade, which includes *Spiroplasma apis*, a pathogen of honey bees (12, 16). The availability of the *S. turonicum* complete genome sequence will facilitate studies to elucidate the evolutionary biology of spiroplasmas.

### Nucleotide sequence accession numbers.

This genome project has been deposited in GenBank under the BioProject ID PRJNA291635, BioSample accession no. SAMN03948565, and GenBank accession no. CP012328.
